# Sequential TAV-in-TAV Resolves LVOT Pseudoaneurysm-Related Paravalvular Leak

**DOI:** 10.1016/j.jaccas.2025.106599

**Published:** 2026-01-21

**Authors:** Zeryab A. Khan, Steven J. Yakubov, Laura D. Flannery, Carlos E. Sanchez

**Affiliations:** OhioHealth Riverside Methodist Hospital, Columbus, Ohio, USA

**Keywords:** aortic valve, cardiovascular disease, stenosis

## Background

Management of severe aortic stenosis (AS) with concomitant left ventricular outflow tract (LVOT) aneurysm or pseudoaneurysm is controversial, with limited evidence guiding optimal transcatheter strategies. Among the limited number of reported cases, valve selection, implantation depth, and sealing of the LVOT aneurysm or pseudoaneurysm vary.

## Case

An 84-year-old man with coronary artery disease status post coronary artery bypass grafting (2001), degenerative mitral regurgitation status post MitraClip (2019), and a membranous ventricular septal defect status post surgical repair (1963) presented with New York Heart Association functional class II dyspnea. Transthoracic echocardiography showed a calcified trileaflet aortic valve, left ventricular ejection fraction of 35%, aortic valve area of 0.8 cm², mean transvalvular gradient of 23 mm Hg, peak aortic jet velocity of 3.2 m/s, and stroke volume index of 23 mL/m², consistent with classical low-flow, low-gradient AS. Computed tomography for transcatheter aortic valve replacement planning revealed a calcium score of 3,560 Agatston units, an annular perimeter of 99.4 mm, a perimeter-derived diameter of 31.6 mm, an annular area of 751.8 mm², an area-derived annular diameter of 31.4 mm, right and left coronary heights of 23.1 mm and 15.3 mm, sinus of valsalva diameters of >33 mm, and a 27 × 14-mm saccular left ventricular outflow tract pseudoaneurysm beneath the right coronary cusp with a 14 × 11-mm ostium. This was likely due to chronic degenerative changes secondary to chronic hemodynamic stress, fibrotic remodeling, or subclinical endocarditis.

## Decision-Making

**Figure 1:**
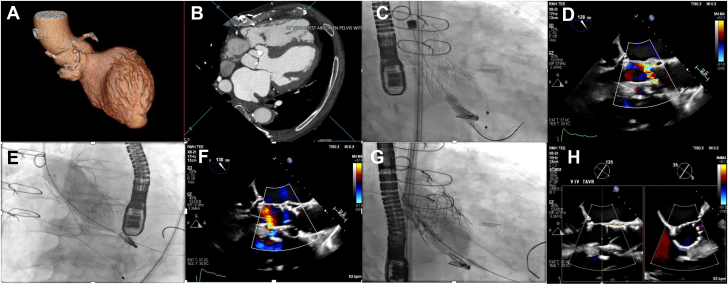
Multimodality Imaging and Procedural Sequence in Transcatheter Aortic Valve-in-Valve Replacement With Concomitant LVOT Pseudoaneurysm Sealing (A) Three-dimensional computed tomography reconstruction demonstrating a saccular left ventricular outflow tract (LVOT) pseudoaneurysm beneath the right coronary cusp. (B) Coronary computed tomography angiography revealing a membranous pseudoaneurysm. (C) Deployment of a 34-mm Evolut Fx + at a low implantation depth. (D) TEE with color Doppler confirming a severe eccentric peri-transcatheter aortic valve (TAV) leak post-Evolut Fx + implantation. (E) Postdeployment balloon valvuloplasty and resultant foreshortening of the 34-mm Evolut Fx+. (F) Peri-TAV leak and incomplete sealing of the LVOT pseudoaneurysm on transesophageal echocardiography. (G) Deployment of the 29-mm Sapien 3 Ultra Resilia within the index valve. (H) TEE long-axis and short-axis views illustrating resolution of paravalvular leak and complete pseudoaneurysm exclusion after TAV-in-TAV implantation.

Given the risk of pseudoaneurysm rupture, thromboembolism, and aortic cusp prolapse, a 34-mm Evolut Fx+ (Medtronic) was selected for its longer sealing skirt, with the intention of covering the pseudoaneurysm through lower implantation depth. A severe eccentric peri-transcatheter aortic valve (TAV) jet was noted after the index TAV deployment, most likely due to low implantation depth with suboptimal annular sealing. Consequently, postdeployment balloon valvuloplasty was performed using a 26- × 4.5-mm True balloon (Bard Peripheral Vascular Inc), which foreshortened the TAV, compromised LVOT pseudoaneurysm sealing, and produced an eccentric peri-TAV jet. A 29-mm Sapien 3 Ultra Resilia (Edwards Lifesciences) was then implanted within the index TAV. This short-in-tall TAV-in-TAV strategy resolved the peri-TAV leak and successfully sealed both the annulus and pseudoaneurysm via the neoskirt (Figure 1 and Video 1).

## Conclusions

In cases of severe AS with concomitant LVOT aneurysm or pseudoaneurysm, exclusion of the aneurysmal defect is critical. This case demonstrates a feasible bailout using a short-in-tall TAV-in-TAV configuration when initial valve deployment fails and paravalvular leak occurs. Planning for low implantation depth with the index TAV carries an increased risk of pacemaker requirement, mitral valve impingement, and the potential need for bailout TAV-in-TAV for skirt leak as demonstrated in this case and should be pursued only when no alternative options exist.

## Funding Support and Author Disclosures

The authors have reported that they have no relationships relevant to the contents of this paper to disclose.

## Appendix

Video 1 Adapted From a Case Originally Presented at Cardiovascular Innovations 2025

